# Modeling Frequency of Injuries per Vehicle Crash in Gurage Zone, Southern Ethiopia

**DOI:** 10.4314/ejhs.v31i1.12

**Published:** 2021-01

**Authors:** Biru Mohammed Derese, Dumga Kassahun Trueha

**Affiliations:** 1 Department of Statistics, College of Natural and Computational Sciences, Wolkite University, Ethiopia; 2 Department of Statistics, College of Natural and Computational Sciences, Wolkite University, Ethiopia

**Keywords:** Frequency of injuries, Negative Binomial Regression Model, Traffic accident

## Abstract

**Background:**

Traffic accident is the most serious problem in developing countries like Ethiopia, which is among the leading cause of death with the highest increasing rate from year to year in Ethiopia. This research aimed to identify the associated factors on the frequency of injuries per vehicle crash in Gurage zone.

**Methods:**

A retrospective study was conducted to identify the contributing factors of a number of injuries per accident. The data were collected from all traffic control and investigation office of 13 Woredas (Districts) for the past five consecutive years from 2013 to 2017. Negative Binomial Regression model was employed to identify the associated factors that affect the number of injuries per accident.

**Results:**

A total of 334 accidents recorded in the last five years from 2013 to 2017 in Gurage zone. Two hundred eight three (84.73%) of the accidents were caused 610 number of injuries. The significantly associated factors of frequency of injuries per road traffic accidents were Drivers' Age (IR: 0.9813; CI: 0.9664 - 0.9962), Drivers' Sex: Female (IR : 1.6386; CI : 1.2176 - 2.0596), Drivers' vehicles ownership: Hired (IR: 1.4216; CI: 1.1697 - 1.6735) and non-drivers' related variables, like weather condition: Rainy (IR: 1.6041; CI: 1.2552 - 1.9529), road shape: street-square (IR: 1.7421 ; CI: 1.1908 - 2.2934) and vehicle type: Isuzu (load)(IR: 1.6845; CI : 1.2592 - 2.1098) Minibus (IR: 2.7253; CI 2.3129 - 3.1377).

**Conclusions:**

This study found that, Driver's related factors: Driver's Age, Sex, Drivers' vehicle ownership, and non-drivers' related variables: Weather condition, Road shape, and Vehicle type were identified as significantly associated factors on the frequency of injuries per vehicle crash in Gurage Zone.

## Introduction

Traffic accidents are among the leading cause of fatalities and injuries that bring serious social, economic, and public health problems of all over the world (1). World Health Organization (WHO) defines a transport accident as “any accident involving a device designed primarily for or being used at the time conveying persons or goods from one place to another” (1). Over the years, traffic accidents have increased at an alarming rate, especially, in developing countries like Ethiopia. According to WHO, 2013 report road traffic-related deaths in Ethiopia reached 22,786 or 2.77% of the total deaths in the country, with over 100 fatalities per 10,000 vehicles (2). The trend is further corroborated in a recent study, which showed that traffic-related accidents make one of the country's highest fatality rates per vehicle in the world. As a comparison, the fatality rate in Kenya and the United Kingdom is about 19 and 2 per 10,000 vehicles, respectively (2–3).

Ethiopian Federal Transport Authority reports that 2017 traffic accidents were the cause for 5,118 people death, 7,754 seriously injuries and 7,775 light injuries across the country (5). However, Ethiopia has one of the lowest per capita car proprietorships in the world but six times more likely traffic accidents were occurring in the country as compared to the USA (5). Even though road traffic accident is an enormous cause of life lose, economically the consequences of traffic accidents are extremely overwhelming our citizen property.

For the last fifteen years, the total road network of Ethiopia has significantly increased from 31,554 km with a road density of 0.50 per thousand people and 28.69 per thousand square km in the year 2000 to 99,522 km with a road density of 1.1 km per thousand people and 90.5 km per thousand square km in the year 2014 (6,7). Even though newly constructed roads were increased, traffic accidents are increasing and road fatalities are growing each year.

Gurage zone is found in the region of Southern Nation and Nnationalities people of Ethiopia. This region has a share of more than 9% of traffic accidents contribution to the country-level in Ethiopia (7,8). The limited government-based annual report was produced on material damage and total injuries types. The report did not incorporate detail of scientific analysis. Therefore, in this research, we attempted to identify the associated factors of the frequency of injuries recorded per accidents in Gurage Zone.

## Methods

**Study setting and design**: The study was conducted in Gurage Zone found in South Nation and Nationalities People of Ethiopia regional state. The zone had 13 woredas (districts).

A retrospective study was conducted on relevant traffic control and investigation Department. Police office documented reports obtained from thirteen Woredas' (districts') police stations found in the zone. The study included documented reports from July 2013 to June 2017. A quantitative method was employed.

**Study population and variables**: Accidents are recorded by traffic police daily. This study is based on secondary data obtained from traffic control and investigation department in the Gurage from July 2013 to June 2017. All vehicle accidents and accident victims registered at the traffic control and investigation from July 2013 to June 2017 were included in the study. The dependent variable is the number of injuries per accident (frequency of injuries per crash). The independent variables include variables related to driver and vehicle characteristics, like gender, age, vehicle type, driver-vehicle relationship, place of the accident, weather condition at the time of the accident, road shape of the accident place, road surface type, and Time of accidents (11).

**Sample size and sampling techniques**: All vehicle accidents and number of injuries per vehicle crash registered at the traffic control and investigation of Gurage zone documented from July 2013 to June 2017 were included in the sample based up on the availability and objective of this study.

**Data collection process and data quality control**: Data were collected from the thirteen districts of traffic control and investigation police stations found in the zone. The data collection was performed using a checklist that was prepared based on the traffic control and investigation registry format, which were conducted in each district. The data collection tool was prepared in English and translated into Amharic and back into English to ensure its consistency. Before the actual data collection, training was given to the data collectors and supervisors. The incomplete data which affects the outcome of the study was excluded. Data collectors were supervised daily for the completeness and consistency of collected data. The data entry was made on Epi Data Version 3.5.1 to minimize data entry errors.

**Data processing and analysis**: The data collected from the thirteen districts of traffic control and investigation police stations in Gurage was entered and cleaned using Epi-info Version 3.5.1 and then, analyzed by using SPSS version 23. The results were presented by using descriptive statistics (proportion, cross-tabulation, and graphs). To identify the associated factors of a number of injuries per accident Negative Binomial Regression Model was employed and reported in terms of relative risk (RR), confidence interval and parameters value at 95% level of significance (*α* = 0.05).

This study was based on the number of injuries per vehicle crashes, assumed to follow the Poisson distribution. In Poisson regression, it is assumed that the dependent variable (number of occurrences of an event) has a Poisson distribution (12). The log link function of Poisson regression is given as:

$$\log(\lambda )=\beta_{0}+\sum_{i=1}^{m}\beta_{i}X_{i}+\varepsilon_{i}$$

where the log of λ (mean and variance of Poisson distribution) is assumed to be a linear function of the independent variables. *β*_0_ is the intercept of the model, *β*_*i*_'s are the coefficients (parameters estimated by maximizing the likelihood function) of independent variables, *X*_*i*_'s is the *m* number of independent variables and *ε*_*i*_ is the error term.

In the presence of over-dispersion (when the variance exceeded the mean of the response variable), Negative Binomial regression is an alternative approach to model the overdispersion count data (12).

The Negative Binomial model has variance = λ + kλ^2^ where k ≥ 0 is the dispersion parameter, when k=0, the negative binomial distribution reduces to Poisson. Therefore, in testing overdispersion, the null hypothesis is H_0_: k=0 and the alternative hypothesis is Ha: k>0. The test can be done by the deviance of a model (12,13).

The goodness of fit tests, for the model is evaluated by two chi-square tests of Pearson statistic and deviance statistic procedure. Model Selection and Comparison also applied using AIC and BIC (12).

## Results

Among 334 vehicles crashes, 283(84.7%) of accidents were caused a total of 610 number of injuries and 51(15.3%) of accidents were free of any number of injuries. The majority accidents 317(94.91%) were occurred by male drivers, and female drivers were involved in lowest number of accidents 17(5.09%). The number of injuries status, at least one injury per accident, was highest, i.e. 181(64.0%) for age ranges of 18–30 years old drivers and greater than 50 years old drivers were involved in lowest 5(1.8%) number of injuries status, at least one injuries per accident. The majority number of injuries status, at least one injury per accident, occurred in sunny weather conditions (52.3%) followed by windy and rainy weather conditions (19.8% and 18.8%). Among the seven types of vehicles, Minibus and Isuzu were involved in the highest frequency of injuries (31.8% and 29.7%). In relation to the place of accident, the highest frequency of injuries was (67.1%) recorded in the rural area. Around 78.8% of the number of injuries occurred on the asphalt road surface. Regarding the driver's education level, respondents with secondary school education were responsible for the largest share of injuries (38.86%) followed by junior education level (31.44%) (Table 1).

**Table 1:** Socioeconomic and demographic related variables by number of injuries status per accident

Variables	Categories	Number of Injuries Status Per Accident				Total

		Zero Injury		At Least One		

		Count	%	Count	%	
Sex of the Denver	Female	1	2.0%	16	5.7%	17
	Male	50	98.0%	267	94.3%	317
Age by Category	18–30	22	43.1%	181	64.0%	203
	31–50	29	56.9%	97	34.3%	126
	>50	0	0.0%	5	1.8%	5
Weather Condition at The Time	Sunny	32	62.7%	148	52.3%	180
of Accident	Cloudy	6	11.8%	27	9.5%	33
	Rainy	6	11.8%	52	18.4%	58
	Windy	7	13.7%	56	19.8%	63
Time of Accident	Morning	13	25.5%	88	31.1%	101
	Afternoon	26	51.0%	129	45.6%	155
	Night	12	23.5%	66	23.3%	78
Vehicle Driver Relationship	Hired	34	66.7%	195	68.9%	229
	Owen	17	33.3%	88	31.1%	105
Road Shape of The Accident	X-Shape	0	0.0%	28	9.9%	28
Place	Street-square	1	2.0%	8	2.8%	9
	T- Shape	2	3.9%	23	8.1%	25
	Curved	10	19.6%	43	15.2%	53
	Straight	33	64.7%	161	56.9%	194
	Escarp	5	9.8%	20	7.1%	25
Place of Accident	Rural	35	68.6%	190	67.1%	225
	Urban	16	31.4%	93	32.9%	109
Road Surface Type	Grabel	23	45.1%	60	21.2%	83
	Asphalt	28	54.9%	223	78.8%	251
Type of Vehicle	Minibus	8	15.7%	90	31.8%	98
	Isuzu(loading)	15	29.4%	84	29.7%	99
	Sino-track	13	25.5%	27	9.5%	40
	Motor-Cycle	2	3.9%	25	8.8%	27
	Autobus and Isuzu (People)	0	0.0%	23	8.1%	23
	Bajaj	5	9.8%	9	3.2%	14
	Toyota-Pickup and Automobile	7	13.7%	20	7.1%	27
	Long- Vehicle	1	2.0%	5	1.8%	6
Education Level of Drivers	Elementary	10	19.6%	61	21.7%	71
	Junior	14	27.5%	89	31.7%	103
	High School	23	45.1%	110	39.1%	133
	Preparatory	2	3.9%	9	3.2%	11
	Certificate and Above	2	3.9%	12	4.3%	14
District of the Accident Place	Abeshege	2	3.90%	67	23.67%	69
	Cheha	5	9.80%	25	8.80%	30
	Kebena	1	2.00%	18	6.40%	19
	Meskan	8	15.70%	60	21.20%	68
	Enemorina Eaner	16	31.40%	26	9.20%	42
	Muhur Na Aklil	0	0.00%	2	0.70%	2
	Mareko	10	19.60%	11	3.90%	21
	Ezha	0	0.00%	19	6.70%	19
	Kohir Gedbano	1	2.00%	2	0.70%	3
	Endiguagn	1	2.00%	2	0.70%	3
	Geta	0	0.00%	2	0.70%	2
	Sodo	7	13.70%	32	11.30%	39
	Gumer	0	0.00%	17	6.00%	17
**Total**		**51**	**15.3%**	**283**	**84.7%**	**334**

The maximum number of injuries was 22 per accident. Averagely, 1.83 number of injuries were occurred per vehicles crashes (Table 2).

**Table 2:** Descriptive statistics of the dependent variable, number of injuries per accident

	No. Accident	Minimum	Maximum	Sum	Mean	Variance	Skewness	Kurtosis
Number of Injuries per Accident	334	0	22	610	1.83	5.351	3.995	24.359

**Figure 1:**
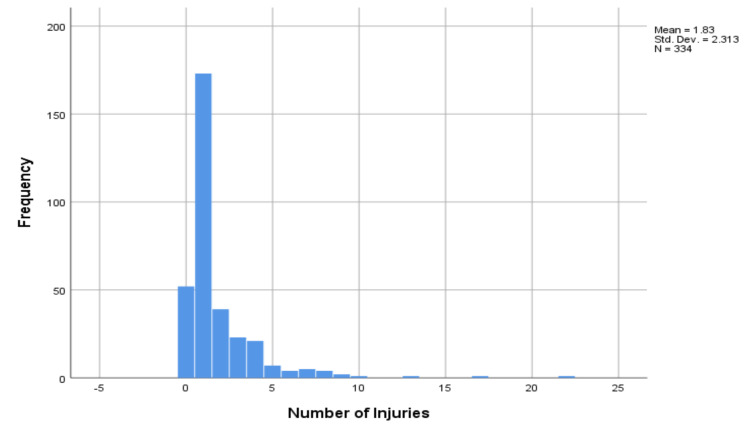
The histogram graph of number of injuries per accident

The distribution of dependent variable number of injuries per accident was right skewed. The Skewness = 3.995 and Kurtosis = 24.359 statistics value from table 1 also support non-symmetrical distribution of number of injuries per accident (Figure 1).

**Figure 2:**
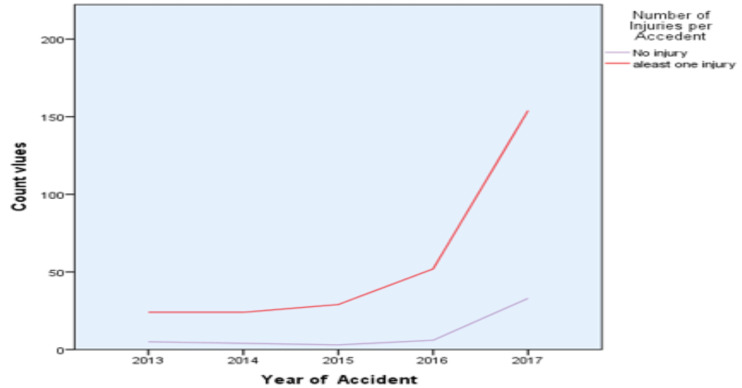
Frequency of injuries per accident by year of accident

The frequency of injuries trend was slightly increased from 2013 to 2017 but frequency of injuries trend has been highly increased from 2015 to 2017 (Figure 2).

**Figure 3:**
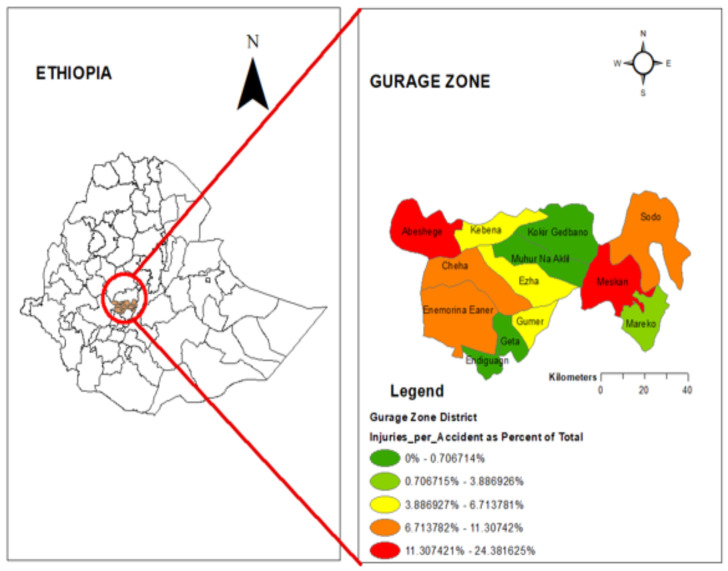
The spatial distribution of the frequency of injuries per accident in Gurage zone.

The spatial distribution of frequency of injuries status per accident was categorized as no injury and at-least one injury per accident of thirteen districts in Gurage zone. The high proportion of at-least one injury status per accident was recorded in Abeshege and Meskan districts (Figure 3).

The mean and variance for the number of injuries per accident were 1.83 and 5.351 respectively. The variance is quite different from the mean which is an indication of overdispersion.

The ratio of Deviance value to its degrees of freedom was 1.4607, and the ratio of Pearson Chi-square value to its degrees of freedom was 1.7356 which is different from one. Thus, it can be concluded that there is statistically significant overdispersion in the data that can lead to the rejection of the assumption of Poisson distribution since the dispersion parameter K is different from zero.

The Negative Binomial model's AIC = 1134.857 and BIC = 1211.0 values were less than that of the Poisson regression model's AIC = 1184.211 and BIC = 1256.623 values. Therefore, to avoid erroneous inferences relating to the factors associated with the frequency of injury per crashes and to fix the problem of dispersion, a Negative Binomial regression model was employed.

Negative Binomial Regression Model was employed to identify the associated factors on number of injuries per vehicle crash. The factors which were statistically significant, associated with number of injuries per accident were age of drivers (IR = 0.9813, 95% CI = 0.9664 - 0.9962, P = 0.018), female drivers (IR = 1.6386, 95% CI = 1.2176 - 2.0596, P = 0.0215), among vehicle types, Isuzu (load) and Minibus (IR = 1.6845, 95% CI = 1.2592 = 2.1098, P = 0.0163 and IR = 2.7253, 95 % CI = 2.3129 -3.1377, P < 0.001) respectively, street-square (IR = 1.7421, 95% CI = 1.1908 - 2.2934, P = 0.0484), Hired Vehicle Driver (IR = 1.4216, 95% CI = 1.1697 - 1.6735, P = 0.0062) and rainy weather conditions (IR = 1.6041, 95% CI = 1.2552 - 1.9529, P = 0.0079 ). Being drive in rainy weather conditions would increase the incident rate of frequency of injuries by 60.41 % more likely than windy weather conditions. However, place of the accident, road surface type and time of accidents had no significant association with number of injuries per accident (Table 3).

**Table 3:** Analysis of parameter estimates using the Negative Binomial Regression Model

Variable	Category	DF	β	SE	Wald	IR	95% C.I. for IR		P-Value
							Lower	Upper	
Sex	Female	1	0.4939	0.2148	5.29	1.6386	1.2176	2.0596	0.0215
	Male (Ref)								
Age		1	-0.018	0.0076	6.19	0.9813	0.9664	0.9962	0.018
Weather	Rainy	1	0.4726	0.178	7.05	1.6041	1.2552	1.9529	0.0079
Condition	windy (Ref)								
Vehicle	Hired	1	0.3518	0.1285	7.5	1.4216	1.1697	1.6735	0.0062
Driver	Owen (Ref)								
Road	Street-square	1	0.5551	0.2813	3.89	1.7421	1.1908	2.2934	0.0484
Shape	Straight (Ref)								
Vehicle	Isuzu(load)	1	0.5215	0.217	5.77	1.6845	1.2592	2.1098	0.0163
Type	Minibus	1	1.0026	0.2104	22.7	2.7253	2.3129	3.1377	<0.001
	Sino-track (Ref)								

DF: Degree of Freedom; SE: Standard Error; CI: Confidence interval; IR: Incidence Rate

## Discussion

This study was framed on analytic approaches of factors that affect the frequency of injuries per vehicle crashes that occurred in Gurage Zone Southern Ethiopia. A total of 334 accidents and 610 frequency of injuries per vehicle crash occurred from July 2013 to June 2017. The majority of vehicle crashes (94.9%) were caused by men. This result is consistent with different findings (10,11). This high impact of males in vehicle crashes might be due to greater immersion of males in driving activities as life careers than females.

The trend of the number of injuries in this study revealed an increasing pattern. Within five consecutive years, the number of the frequency of injury rate increased more than triple through July 2013 (8.7 %) to June 2017 (29%). Similar to this study, in Gedeo Zone, Ethiopia, a cross-sectional study in Dubai and a Lithuania time trends study (18) reported a progressively increasing amount of frequency of injuries per accident, and also the result is similar with other studies too (16,17,20). However, the result shows underestimate in the Gurage zone, Ethiopia. This underestimate result might be due to the area coverage of the target population that was small and not included main cities found in the county. The severity of number of injuries per vehicle crashes in developing countries is much greater than that of developed countries (14,15,19,24). The main reason for the increment of high number of injuries per accidents could be due to the rise in the number of vehicles that is not balanced to road density per person, poor transport conditions, lack of periodically vehicle technical inspections and poor implementation of road safety policy (11). Our study revealed that drivers who completed secondary educational levels caused a large number of frequency of injuries. The finding is similar to some other studies (14,17). In this study, the age of drivers was significantly associated with the frequency of injuries. The age group 18–30 years was more vulnerable to many injuries' occurrence. Similarly, a mortality survey studied in India showed that younger age drivers were one of the causes of a high frequency of injuries per traffic accidents (9–11, 17,21). Based on reports of some study, the possible reason for young drivers has a higher crash risk than older drivers because of the lack of experience, high risk-taking behavior, thrill-seeking, and over-confidence; less tolerance, excess or inappropriate speed (4,11).

This study revealed that vehicle type was a significantly associated variable and the highest number of the frequency of injuries per accident occurred in terms of commercial vehicle type like Minibus taxis and Isuzu (load). This is consistent with other research done in Benin City and Nigeria, a large number of fatal accidents happened by commercial cars like 12 seat capacity taxi (18). The commercial vehicle contribution to the raise of the frequency of injuries per crashes could be due to the fact that their service is primarily involving conveying persons from one place to another. Furthermore, it may be due to lack of maintenance as sufficient as vehicle service year, overloading problems, lack of usage of safety belt, driver's illegal relationships with traffic policies. (4).

The non drivers factors like afternoon and sunny weather conditions are significantly associated with the frequency of injuries per accident and responsible for the highest frequency of injuries per accident which is similar to a study conducted in hospital-based analysis in Tikur Anbessa specialized hospital, Ethiopia (22,23). This might be related to the fact that the majority of vehicles activities were tied to these two situations and that the sunny weather condition covers large period of time throughout the year in the study area.

This study has limitation. It missed some important factors like vehicle speed record, alcohol consumption status, drug intake status, experience, and other factors due to the fact that we used secondary data that was not available or well recorded in the traffic police control department. In this study, incomplete data were not included due to the fact that some of the districts had poor data collection and data management system.

The results of the study show that the number of injuries per accident was statistically associated with variables related to drivers: drivers' age, drivers' sex, drivers' vehicle ownership, and non-drivers' related variables: weather condition, road shape, and vehicle.

The result showed that in the age group of 18–30 years drivers are taking the largest share of a number of injuries. Therefore, the concerned body should give more emphasis while giving a driving license to this age group.

The highest number of injuries were recorded by drivers of the Isuzu (load) and minibus vehicle type. For this reason, the driver should take care of their part in reducing the number of injuries. Moreover, the government should check periodically every technical parts of the vehicle.
